# Use of translational modeling and simulation for quantitative comparison of PF-06804103, a new generation HER2 ADC, with Trastuzumab-DM1

**DOI:** 10.1007/s10928-020-09702-3

**Published:** 2020-07-24

**Authors:** Alison Betts, Tracey Clark, Paul Jasper, John Tolsma, Piet H. van der Graaf, Edmund I. Graziani, Edward Rosfjord, Matthew Sung, Dangshe Ma, Frank Barletta

**Affiliations:** 1 Department of Biomedicine Design, Pfizer Inc, 610 Main Street, Cambridge, MA 02139 USA; 2 Worldwide Research Procurement, Pfizer Inc, Eastern Point Rd, Groton, CT 06340 USA; 3 RES Group, Inc, 75 Second Avenue, Needham, MA 02494 USA; 4 Division of Systems Biomedicine and Pharmacology, Leiden Academic Centre for Drug Research, 2300 RA Leiden, The Netherlands; 5 Present Address: Apertor Labs Inc, 828 Contra Costa Ave, Berkeley, CA 94707 USA; 6 Oncology Research & Development, Pfizer Inc, 401 N Middletown Rd, Pearl River, NY 10965 USA; 7 Present Address: Department of Therapeutic Proteins, Regeneron, Tarrytown, NY 10591 USA; 8 Present Address: Applied Biomath, 561 Virginia Rd, Suite 220, Concord, MA 01742 USA; 9 Department of Biomedicine, Design Pfizer Inc, Design Pfizer Inc, Pearl River, NY 10965 USA

**Keywords:** HER2, Antibody drug conjugate, Translational modeling, Tumor static concentration, Pharmacokinetics, PK/PD, Oncology

## Abstract

A modeling and simulation approach was used for quantitative comparison of a new generation HER2 antibody drug conjugate (ADC, PF-06804103) with trastuzumab-DM1 (T-DM1). To compare preclinical efficacy, the pharmacokinetic (PK)/pharmacodynamic (PD) relationship of PF-06804103 and T-DM1 was determined across a range of mouse tumor xenograft models, using a tumor growth inhibition model. The tumor static concentration was assigned as the minimal efficacious concentration. PF-06804103 was concluded to be more potent than T-DM1 across cell lines studied. TSCs ranged from 1.0 to 9.8 µg/mL (n = 7) for PF-06804103 and from 4.7 to 29 µg/mL (n = 5) for T-DM1. Two experimental models which were resistant to T-DM1, responded to PF-06804103 treatment. A mechanism-based target mediated drug disposition (TMDD) model was used to predict the human PK of PF-06804103. This model was constructed and validated based on T-DM1 which has non-linear PK at doses administered in the clinic, driven by binding to shed HER2. Non-linear PK is predicted for PF-06804103 in the clinic and is dependent upon circulating HER2 extracellular domain (ECD) concentrations. The models were translated to human and suggested greater efficacy for PF-06804103 compared to T-DM1. In conclusion, a fit-for-purpose translational PK/PD strategy for ADCs is presented and used to compare a new generation HER2 ADC with T-DM1.

**Electronic supplementary material:**

The online version of this article (10.1007/s10928-020-09702-3) contains supplementary material, which is available to authorized users.

## Introduction

Human epidermal growth factor receptor 2 (HER2) over-expression in cancer patients is a genetic alteration that promotes cancer cell proliferation and survival, resulting in increased tumor growth and poor clinical outcome in the absence of HER2 targeted therapy [1, 2]. HER2+ cancers account for approximately 20% of all breast cancers [1, 2]. Trastuzumab, a monoclonal antibody (mAb) which specifically targets HER2, has revolutionized treatment as one of the first non-hormonal medicines for breast cancer [3].

ADCs are a targeted therapy for cancer treatment, combining a specific mAb to a tumor antigen linked to a potent cytotoxic agent [4]. The aim for this type of therapeutic is to target the cytotoxic drug to tumor cells, thus maximizing efficacy while minimizing systemic toxicity due to normal tissue exposure. In 2013, the anti-HER2 ADC T-DM1 was approved, offering greater potential efficacy and enhanced survival by conjugation of a cytotoxic payload (DM1) to trastuzumab [5]. However, both trastuzumab and T-DM1 are only efficacious in patients with high HER2 expression and patients are acquiring resistance [6, 7]. As such there remains a need for improved HER2 therapies to reach a broader spectrum of patients and reduce risk of disease recurrence.

PF-06804103 is a new generation HER2 ADC with an auristatin microtubule inhibitor payload (Aur-101) conjugated to an anti-HER2 IgG1 mAb via a site specific mcValCitPABC cleavable linker [8]. Although both PF-06804103 and T-DM1 are anti-HER2 ADCs, they differ in their linker-payloads and their conjugation chemistry, which has a significant effect on their mechanism of action [9–12]. T-DM1 has a maytansine derived payload (DM1) which is linked via a stable thioether linker to native lysines on trastuzumab. The conjugation method results in a heterogeneous mixture of conjugates with an average of 3.0–3.6 drugs per antibody, and a range of 0–6. Upon binding to HER2, T-DM1 undergoes receptor mediated internalization and trafficking from the endosomes to the lysosomes. In the lysosome T-DM1 undergoes proteolytic degradation, which releases the cytotoxic DM1-linker-lysine-metabolite (lysine-MCC-DM1). This metabolite must be actively transported from the lysosome in order to reach its intra-cellular site of action [13].

The payload of PF-06804103 is conjugated to specific cysteines on the anti-HER2 mAb which have been mutated at fixed locations. This results in production of a homogeneous ADC, with a fixed drug to antibody ratio (DAR) of 4.0. PF-06804103 is also internalized upon binding to HER2, and cleavage of the protease linker results in release of the Aur-101 payload in the endosomes. This is sufficiently permeable to diffuse out of the endosomes and into the nucleus. Unlike T-DM1, the permeability of the released payload means it can enter adjacent cells and mediate cell death, a process referred to as bystander effect [14]. This has been demonstrated in xenograft models in mouse where PF-06804103 enables potent tumor activity in non-HER2 amplified breast cancer and heterogeneous low HER2 models, where T-DM1 is ineffective. The site-specific conjugation method used in PF-06804103 should enable greater stability with more consistent efficacy and the bystander effect should enable treatment of patients with more heterogeneous tumors and lower HER2 expression. Differences in linker-payload chemistry of PF-06804103 compared to T-DM1 should also impede mechanisms of resistance specific to lysine-MCC-DM1, including impaired lysosomal degradation or enhanced efflux [15, 16].

In this manuscript, mathematical modeling and simulation is used as a tool to quantitatively compare PF-06804103 and T-DM1, in terms of their PK and efficacy. A modeling-based method is provided to assess efficacious concentration of PF-06804103 and T-DM1 across preclinical cell line xenograft (CLX) and patient derived xenograft (PDX) studies in mouse. A mechanistic TMDD model is applied to account for variation in shed HER2 and to describe T-DM1 non-linearity in patients. A similar model is then used to predict clinical PK for PF-06804103. A fit-for-purpose translational strategy is proposed to predict clinical efficacy in patients.

## Methods

### Compounds

PF-06804103 was synthesized at Pfizer as described [8]. Trastuzumab-maytansinoid conjugate was synthesized at Pfizer and is structurally similar to trastuzumab emtansine (T-DM1) with similar in vitro potency and in vivo efficacy [8]. It is comprised of an anti-HER2 trastuzumab antibody covalently bound to DM1 through a bifunctional linker. Conjugation was conducted as described previously [4].

### Animal studies

All animal studies were approved by the Pfizer Institutional Animal Care and Use Committee according to established guidelines.

### PF-06804103 In vivo mouse and cynomolgus monkey PK studies

PF-06804103 was administered as a single intravenous (IV) bolus dose of 3 mg/kg to female athymic nu/nu mice (n = 4/dose). Blood samples were collected pre-dose and at 0.083, 6, 24, 48, 96 168 and 336 h post dose. PF-06804103 was administered to cynomolgus monkey as multiple IV bolus doses, given every 3 weeks for a total of 3 doses at 3 mg/kg, 6 mg/kg (both n = 3 males, n = 3 females) and at 12 mg/kg (n = 5 males, n = 5 females). Blood samples were collected pre-dose and at 0.083, 6, 24, 72, 168, 336 and 504 h post-dose.

### PF-06804103 assay

Quantitation of ADC (mAb with at least one drug molecule conjugated) concentrations in plasma collected from female athymic nu/nu mice and cynomolgus monkeys following administration of PF-06804103 (or T-DM1) was achieved using Gyrolab™ (Gyros Protein Technologies, Uppsala, Sweden). Isolation and detection of ADC concentrations from biological matrix was carried out with streptavidin coupled micro columns located on Bioaffy™200 compact discs (CDs), an integrated nanoliter scale immunoassay device, within Gyrolab™. Plasma calibration standards, quality control samples and plasma study samples were all diluted to the minimum required dilution (MRD) and loaded onto the CDs. For measurement of ADC, a sheep anti-human IgG (Binding Site, San Diego, CA) reagent was used for capture and an internally generated mouse anti-payload reagent for detection. Fluorescence of analyte was measured using a laser embedded within the workstation. All data was processed using Watson v7.4 LIMS with a 1/Y*2 weighting.

### In vivo mouse xenograft studies

Mouse efficacy studies were completed in 4 CLX models (JIMT-1, BT474 and HCC-1954 derived from breast cancers, N87 derived from gastric cancer) and 4 PDX models (24312 and 144580 derived from breast, 37622 from lung and GA3109 from gastric tumors). Female athymic nude mice (Nude, Stock No: 002019) were obtained from the Jackson Laboratory (Farmington, CT). For the CLX models, nude mice were injected subcutaneously in the flank with suspensions of 1 × 10^6^ N87 cells, 5 × 10^6^ JIMT-1 cells, 5 × 10^6^ HCC-1954 cells or 10 × 10^6^ BT474 cells in 50% Matrigel (BD Biosciences, Franklin Lakes, NJ). For the PDX models, tumor fragments were subcutaneously passaged in vivo from animal to animal in nude mice. Mice were randomized into study groups when tumors reached approximately 150 to 300 mm^3^. Either phosphate buffered saline (PBS, Gibco, Cat#14190-144, as vehicle), PF-06804103, or T-DM1 were administered IV at different doses starting on day 0 for a total of four doses, 4 days apart (Q4d × 4). Dose levels administered in each tumor model are shown in Table 2 for PF-06804103 and Table 3 for T-DM1. Tumors were measured at least weekly with a calibrator (Mitutoyo, Aurora, Illinois) and the tumor mass was calculated as volume = (width × width × length)/2. These studies have been described previously [8].

### PK/PD modeling in mouse

#### Pharmacokinetics of PF-06804103 and T-DM1 in mouse and PF-06804103 in cynomolgus monkey

**Fig. 1:**
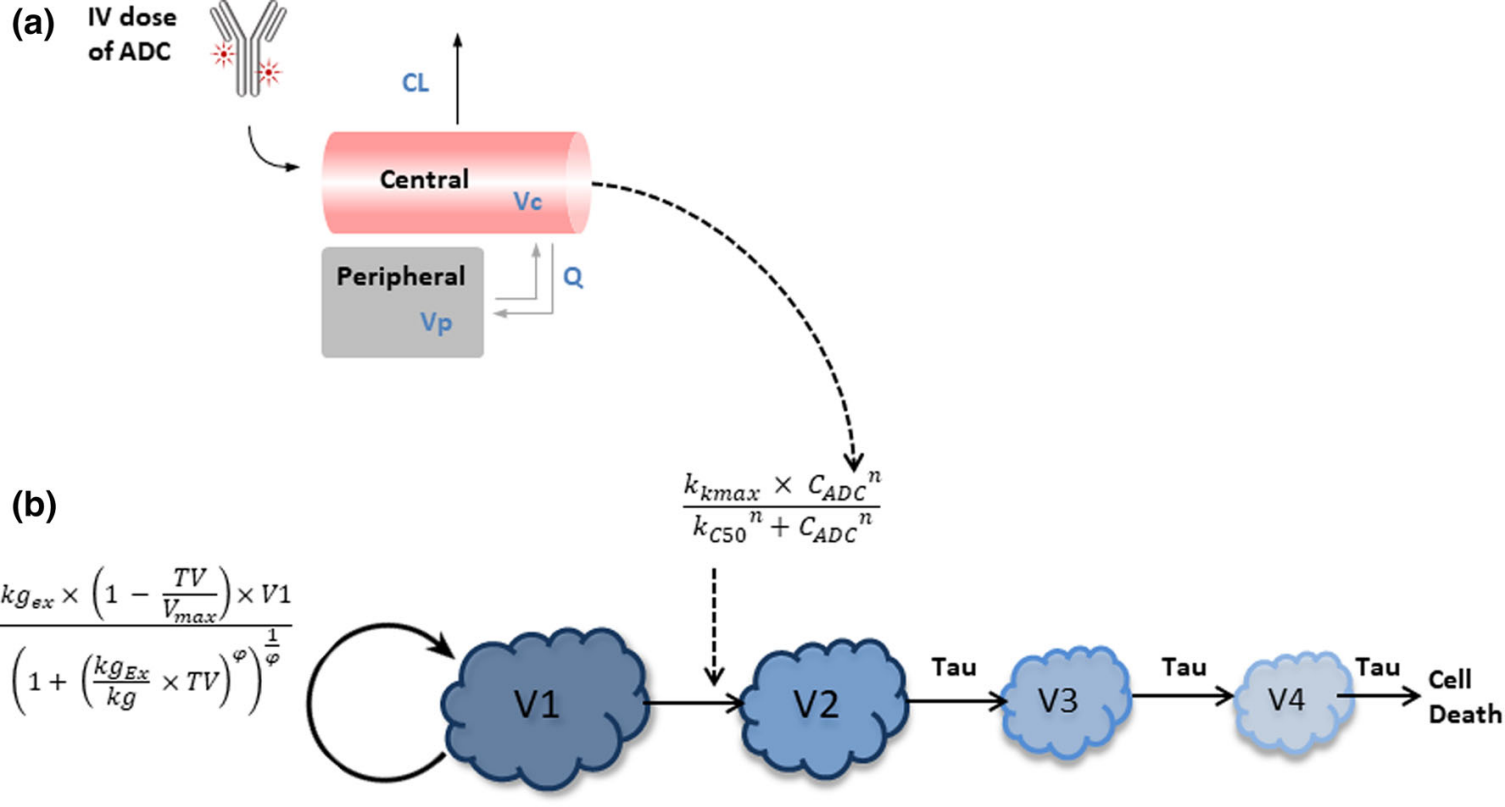
PK/PD model used for the mouse tumor growth inhibition modeling. **a** 2-compartmental linear PK model is linked to **b** a model of tumor growth inhibition. Please refer to Tables 1 and 2 for description of the model parameters

The PK of PF-06804103 in non-tumor bearing mouse following a single IV dose of 3 mg/kg and in cynomolgus monkey following multiple dose IV administration at 3, 6 and 12 mg/kg Q3W × 3 were characterized using a 2-compartment PK model with linear elimination from the central compartment (Fig. 1a). T-DM1 PK in mouse was taken from the literature, where it was linear across the dose range studied (0.3–15 mg/kg) [17].

#### Tumor growth inhibition PK/PD in xenograft mouse as a function of PF-06804103 or T-DM1 concentration

The mouse xenograft PK/PD relationship was established by relating PF-06804103 (or T-DM1) plasma concentration in mouse to measured xenograft tumor size data using a tumor growth inhibition model (Fig. 1b; [18]). The mouse PK parameters derived above were fixed in the subsequent PD modeling of the xenograft mouse data. The presented model is a modified version of the model by Simeoni et al. [19]*.* Briefly, the unperturbed tumor growth was fitted first using individual animal growth data from the vehicle control group, using a logistic model describing linear (*k*_*g*_) and exponential (*k*_*gEx*_) growth. The measured initial tumor volume in each animal was used for the initial conditions (*v0*). V1–V4 are the tumor volume in the growth compartment and three transduction compartments, respectively. *TV is* the total tumor volume (mm^3^). The inter-individual variability of the growth parameters and the maximum tumor volume (*V*_*max*_) obtained from the unperturbed growth model were then fixed in the simultaneous estimation of growth and drug effect parameters from the complete tumor volume data set. *τ* is the transduction time, *k*_*kmax*_ is the maximum kill rate, *kc*_*50*_ is the concentration of PF-06804103 or T-DM1 in the plasma at half the maximal kill rate, *n* is the hill co-efficient and ψ is the constant for switching from exponential to linear growth patterns. ψ was fixed to a value of 20 in all cases [19]. *C*_*ADC*_ is equivalent to free ADC plasma concentration, as no shed HER2 ECD was detected in mouse. Equations 1–6 describe the tumor growth inhibition modeling.

1 $${k}_{kill}= \frac{{k}_{kmax}\times {{C}_{ADC}}^{n}}{{{kc}_{50}}^{n}+ {{C}_{ADC}}^{n}}$$

2 $$\frac{d{V}_{1}}{dt}=\frac{{k}_{gEx} \times \left(1-\frac{TV}{{V}_{max}}\right){\times V}_{1}}{{\left(1+{\left(\frac{{k}_{gex}}{{k}_{g}} \times TV\right)}^{\psi }\right)}^{1/\psi }}-{k}_{kill} \times {V}_{1}$$

3 $$\frac{d{V}_{2}}{dt}={k}_{kill}\times {V}_{1}-\frac{{V}_{2}}{\tau }$$

4 $$\frac{d{V}_{3}}{dt}=\frac{{V}_{2}-{V}_{3}}{\tau }$$

5 $$\frac{d{V}_{4}}{dt}=\frac{{V}_{3}-{V}_{4}}{\tau }$$

6 $$TV={V}_{1}+{V}_{2}+{V}_{3}+{V}_{4}$$

Initial conditions: *TV(t* = *0)* = *V1(t* = *0)* = *v0; V2(t* = *0)* = *V3(t* = *0)* = *V4(t* = *0)* = *0.*

### Calculation of TSC

TSC was defined as the concentration of PF-06804103 or T-DM1 where tumor growth and death rates are equal and tumor volume remains unchanged. This PK/PD derived parameter combines the growth pattern information and the drug effect, providing insight on the efficacy of the ADC. See Eq. 7 for TSC calculation. An 80% confidence interval on TSC was calculated using parametric bootstrap by resampling from the estimated parameters using a log-normal distribution.

7 $$TSC= \frac{{k}_{gEx }\times {{k}_{C50}}^{n} \times \left(1- \frac{{V}_{0}}{{V}_{max}}\right)}{{\left({k}_{kmax }\times {\left(1+{\left(\frac{{kg}_{Ex}}{kg} \times {V}_{0}\right)}^{\varphi } \right)}^{\frac{1}{\varphi }}- {kg}_{Ex} \times \left(1 - \frac{{V}_{0}}{{V}_{max}}\right)\right)}^\frac{1}{n}}$$

#### Modeling

All modeling was performed using Monolix software v2016 (Paris, France) using the solver for stiff ordinary differential equations. The quality of the model fitting was assessed using:

Diagnostic plots: (a) plots of observations versus population/individual predictions and comparison with line of unity, (b) plots of weighted residuals versus time/concentration and check for systematic deviation from zero, (c) visual predictive checks of observations and predictions for all individuals at each dose level to check for goodness of fit [20].

Diagnostic criteria: (a) reasonable precision of the parameter estimates (RSE/CV%) (b) lack of correlation between model predicted parameters (<0.95) (c) lack of shrinkage (η-) as a check for model over-parameterization (<40%) (d) reduction in objective function values and/or Akaike and Schwarz criterion for model comparison (e) Condition numbers (included in Tables 2 and 3). As a rule of thumb, condition number should be less than 10^Npar^ where Npar is the number of parameters estimated in the model for a well-defined model with respect to the information in the data [21]. However, as with all these diagnostic checks, the condition number cannot be taken in isolation, and must be interpreted with respect to all the other criteria.

### Clinical PK predictions

To predict the human PK for PF-06804103 a TMDD model [22] was constructed, incorporating binding to serum HER2 and subsequent elimination of the complex into a standard 2-compartmental PK model. The extracellular domain (ECD) of HER2 is known to shed from the trans-membrane receptor at high levels in the target patient population (metastatic breast cancer) [23]. The presence of shed target is hypothesized to drive non-linear clearance of T-DM1. The TMDD model was initially used to fit the non-linear PK of T-DM1 observed in patients at doses administered in the clinic [24]. The model describes linear, catabolic clearance of T-DM1 (CL), as well as shedding of HER2 ECD (*kshed*), degradation of HER2 ECD (*kdeg*), binding of T-DM1 to HER2 ECD (K_D_, *k*_*on*_ and *k*_*off*_) and elimination of the complex (*kel*_ADC-ECD_). The model structure is shown in Fig. 3. The concentration of HER2 ECD was initially set to 20 ng/mL (0.2 nM) which is above the normal upper limit in healthy females (15 ng/mL), and above the median in metastatic breast cancer patients [25]. To improve the individual fit at each dose level, ECD concentration was varied between 16 and 28 ng/mL. Patients with higher ECD concentrations had more rapid clearance due to TMDD and varying the ECD concentrations enabled better description of the slope of the PK curves observed. The *k*_*on*_ and K_D_ of T-DM1 were fixed in the model at 61.3 nM^−1^ day^−1^ and 0.1 nM, respectively [26]. The binding of T-DM1 to HER2 was assumed to be the same for HER2 ECD and transmembrane domain. *kshed, kdeg* and *kel*_ADC-ECD complex_ were all estimated in the model fitting process.

The model was then applied to predict the human PK of PF-06804103. The 2-compartment linear parameters were scaled from cynomolgus monkey population PK parameters using allometric scaling exponents of 1 for volumes and 0.9 for clearance parameters [27]. The binding of PF-06804103 to HER2 was incorporated into the model and assumed to be the same for HER2 ECD and transmembrane domain. The rate of HER2 shedding, HER2 degradation and the elimination of the complex were set to that estimated from the model used to fit T-DM1 PK data in patients. To investigate the impact of HER2 ECD concentrations on PK/PD, simulations were also performed at low (2 ng/mL) and high (750 ng/mL) HER2 ECD concentrations, representing the range of concentrations reported across 78 healthy females and 100 patients with metastatic breast cancer [25]. Equations 8-11 describe the TMDD modeling. *C*_*ADC*_ is the free ADC concentration in the central compartment (nM), *C*_*ADC_per*_ is the ADC concentration in peripheral (i.e. tissue) compartment (nM), ${C}_{ECD}$ is the HER2 ECD concentration (nM) and ${C}_{ADC\_ECD}$ is the ADC- ECD complex (nM). *In(t)* is the infusion rate of the drug in nM/h, based on a MW of the drug of 150 kDa; the infusion duration was 1 h.

Equations:

8 $$\frac{d{C}_{ADC}}{dt} =In \left(t\right)-\left(\frac{CL}{Vc}\times {C}_{ADC}\right)-\left(\frac{Q}{Vc}\times {C}_{ADC}\right)+\left(\frac{Q}{Vp}\times {C}_{ADC\_per}\times \frac{Vp}{Vc} \right) -\left(kon\times {C}_{ADC}\times {C}_{ECD} \right)+\left(koff \times {C}_{ADC\_ECD}\right)$$

9 $$\frac{d{C}_{ADC\_per} }{dt}=\left(\frac{Q}{Vc}\times {C}_{ADC}\times \frac{Vc}{Vp}\right)-\left(\frac{Q}{Vp}\times {C}_{AD{C}_{per}}\right)$$

10 $$\frac{d{C}_{ECD} }{dt}= {kshed}_{HER2\_ECD}- \left({kdeg}_{HER2\_ECD} \times {C}_{ECD}\right)- \left(kon\times {C}_{ADC}\times {C}_{ECD}\right)+\left( koff \times {C}_{ADC\_ECD}\right)$$

11 $$\frac{d{C}_{ADC\_ECD}}{dt}= \left(kon\times {C}_{ADC}\times {C}_{ECD}\right)- \left(koff \times {C}_{ADC\_ECD}\right)-\left({kel}_{ADC\_ECD} \times {C}_{ADC\_ECD}\right)$$

Initial conditions: $${C}_{ADC}\left(t=0\right)={C}_{AD{C}_{per}}\left(t=0\right)={C}_{AD{C}_{ECD}}\left(t=0\right)=0;{C}_{ECD}\left(t=0\right)=0.206 nM$$

### Clinical PK/PD predictions

The PD parameters estimated from mouse xenograft studies (Table 2 for PF-06804103 and Table 3 for T-DM1) were integrated with the predicted human PK parameters (Table 4) to project clinical efficacy (tumor regression) following Q3w×4 dosing of PF-06804103 at 1 mg/kg and T-DM1 at 3.6 mg/kg (clinical dose). It was assumed that mouse PD parameters translate directly to human (including initial tumor volumes). Due to the growth rate difference between xenograft models and clinical tumors, the predictions that achieve stasis using mouse xenograft PD parameters are assumed to be minimally efficacious in human, achieving greater than stable disease [18]. This method has been tested previously for T-DM1 and resulted in accurate predictions of efficacious dose in the clinic [18].

## Results

### PF-06804103 PK in mouse and cynomolgus monkey

To determine the PK/PD relationship in mouse, PK was determined separately following IV administration of PF-06804103 at 3 mg/kg and described using a 2-compartment linear model. To inform PF-06804103 clinical PK predictions, PK was determined in Cynomolgus monkey following multiple dose IV administration at 3, 6 and 12 mg/kg Q3W×3. The PK was linear in monkey across the dose range studied and could be described using a 2-compartment PK model. This was expected as there is no shed HER2 ECD in cynomolgus monkey. The terminal half-life in monkey was approximately 7 days. The 2-compartment model parameters in mouse and Cynomolgus monkey are shown in Table 1.

**Table 1:** Mouse and Cynomolgus monkey PK parameters for PF-06804103

Parameter (unit)^a^	Description	Mouse^b^	Cynomolgus monkey^c^ (CV %)
Vc (mL/kg)	Central compartment volume	61.0	38.1 (3)
CL (mL/day/kg)	Clearance	22.8	7.2 (5)
Vp (mL/kg)	Peripheral compartment volume	56.2	20.2 (7)
Q (mL/day/kg)	Inter-compartmental clearance	35.0	19.2 (18)

^a^Macro-constants conversion to micro-constants: kel = CL/Vc; k12 = Q/Vc; k21 = Q/Vp. ^b^PK of PF-06804103 was determined in mouse following single IV administration at 3 mg/kg. Mean PK values were fitted to a 2-compartment model (no % CV derived). ^c^PK of PF-06804103 was determined in cynomolgus monkey following IV administration on day 1 at 3, 6, or 12 mg/kg. A 2-compartment population PK model was used to fit the cynomolgus monkey data with *Omega V1* = 0.0589 (48), *Omega CL* = 0.22 (16) and proportional error = 0.131 (7)

### PF-06804103 and T-DM1 PK/PD relationship in mouse tumor xenograft models

The ability of PF-06804103 and T-DM1 to regress tumors was studied in a range of CLX and PDX experimental mouse tumor models. The mouse PK parameters were integrated with the tumor volume data over time following different dose levels of drug to describe the ADC concentration versus response relationship. The model described the differences in growth rates observed across the tumor models. It described the delay between drug administration and tumor cell killing due to signal transduction. The different tumor models had varying susceptibilities to drug effect which are observed as differences in potency (*kc*_*50*_) and kill rate (*k*_*kmax*_) of PF-06804103 and T-DM1 across models. PD parameters determined for PF-06804103 in 7 mouse xenograft models (3 CLX and 4 PDX) are shown in Table 2 and goodness of fit plots are shown in Supplementary Fig. 1. PD parameters for T-DM1 across 3 CLX models in mouse are shown in Table 3.

**Table 2:** PF-06804103 estimated PD model parameters (CV %) and derived TSC values [80% confidence intervals] in 3 CLX and 4 PDX mouse models

Parameter (unit)	Description	JIMT-1 CLX (Breast)	N87 CLX (Gastric)	BT474 CLX (Breast)	24312 PDX (Breast)	37622 PDX (NSCLC)	144580 PDX (Breast)	GA-3109 PDX (Gastric)
Doses (mg/kg)	IV q4d × 4	0, 0.25, 0.5, 1	0, 0.3, 1, 3	0, 0.5, 1.5	0, 1.5, 3, 6	0, 0.3, 1, 3	0, 1.5, 3, 6	0, 1, 3
*k*_*g,Ex*_ (day^−1^)	Exponential growth rate	0.0883 (8)	0.068 (8)	0.0442 (65)	0.023 (12)	0.0559 (9)	0.0461 (8)	0.115 (6)
*k*_*g*_ (mm^3^ day^−1^)	Linear growth rate	47.5 (23)	26.8 (14)	78.5 (23)	24.4 (15)	68.4 (22)	395 (65)	57.2 (9)
*V*_*max*_ (mm^3^)	Maximum growth rate	4.08E+03 (15)	4.60E+03 (20)	5.28E+03 (23)	5.00E+03 (–)	3.84E+03 (13)	5.92E+03 (16)	7.07E+03 (26)
*τ* (day)	Transduction time	2.23 (6)	2.54 (6)	3.04 (16)	1.66 (1)	3.32 (5)	9 (5)	5.81 (2)
*k*_*k,max*_ (day^−1^)	Maximum kill rate	0.703 (9)	0.15 (5)	0.998 (209)	0.721 (0)	0.362 (13)	0.516 (13)	1.24 (2)
*kc*_*50*_ (µg mL^−1^)	Concentration at half maximal kill rate	10.6 (9)	1.24 (16)	31.5 (236)	15.8 (5)	4.19 (19)	25.8 (16)	14.7 (6)
*n*	–	2.4 (12)	1 (-)	1 (-)	2.6 (-)	1.3 (7)	2.4 (21)	2.5 (6)
*ψ*	–	20 (-)	20 (-)	20 (-)	20 (-)	20 (-)	20 (-)	20 (-)
*Omega k*_*g,Ex*_		0.401 (12)	0.271 (-)	2.59 (17)	0.373 (-)	0.372 (-)	0.359 (-)	0.25 (-)
*Omega k*_*g*_		1.23 (14)	0.666 (-)	0.717 (23)	0.0441 (-)	0.789 (-)	1.3 (-)	0.316 (-)
Additive error		13.4 (12)	34.9 (7)	106 (6)	19.1 (6)	35.8 (3)	63.4 (4)	18.3 (4)
Proportional error		0.118 (6)	0.055 (12)	-	0.227 (6)	0.0755 (8)	0.0648 (11)	0.188 (5)
Condition number		2.4E+03	1.5	2.7E+03	2.7E+03	87	85	2.7E+04
TSC (µg mL^−1^) [80% CI]	Tumor static concentration	4.8 [4.2, 5.5]	1.0 [0.8, 1.4]	3.0 [-]	4.3 [3.8, 4.6]	1.2 [0.8, 1.5]	9.8 [8.0, 12.0]	5.8 [5.3, 6.2]

**Table 3:** T-DM1 estimated PD model parameters (CV %) and derived TSC values [80% confidence intervals] for 3 CLX models in mouse (N87, BT474 and HCC-1954)

Parameter (unit)	Description			JIMT-1 CLX (Breast)	N87 CLX (Breast)	BT474 CLX (Gastric)	144580 PDX (Breast)	HCC-1954 CLX (Breast)
Doses (mg/kg)	IV q4d × 4			6	0, 1, 3, 10	0, 1, 3, 10	6	0, 0.3, 1, 3
*k*_*g, Ex*_ (day^−1^)	Exponential growth rate			No response	0.0732 (11)	0.0575 (46)	No response	0.0918 (8)
*k*_*g*_ (mm^3^ day^−1^)	Linear growth rate				37.9 (17)	77.4 (20)		40.7 (6)
*V*_*max*_ (mm^3^)	Maximum tumor volume				4.22E+03 (18)	5.28E+03 (23)		3.18E+03 (27)
*τ* (day)	Transduction time				1.36 (16)	2.4 (7)		1 (8)
*k*_*k, max*_ (day^−1^)	Maximum kill rate				0.405 (38)	1.38 (91)		0.319 (7)
*kc*_*50*_ (µg mL^−1^)	Concentration at half maximal kill rate				131 (48)	311 (110)		8.63 (10)
*n*	–				1 (-)	1.01 (4)		1.5 (-)
*ψ*	–				20 (-)	20 (-)		20 (-)
*Omega k*_*g,Ex*_					0.47 (-)	2.26 (15)		0.371 (-)
*Omega k*_*g*_					0.781 (-)	0.917 (16)		0.274 (-)
Additive error					66.8 (6)	30 (-)		14.4 (10)
Proportional error					0.0727 (12)	0.157 (5)		0.0754 (8)
Condition number					250	9E+05		27
TSC (µg mL^−1^) [80% CI]	Tumor static concentration				29 [13, 67]	14 [2.4, 57]		4.7 [4.0, 5.6]

### Comparison of PF-06804103 and T-DM1 efficacy using TSC values

**Fig. 2:**
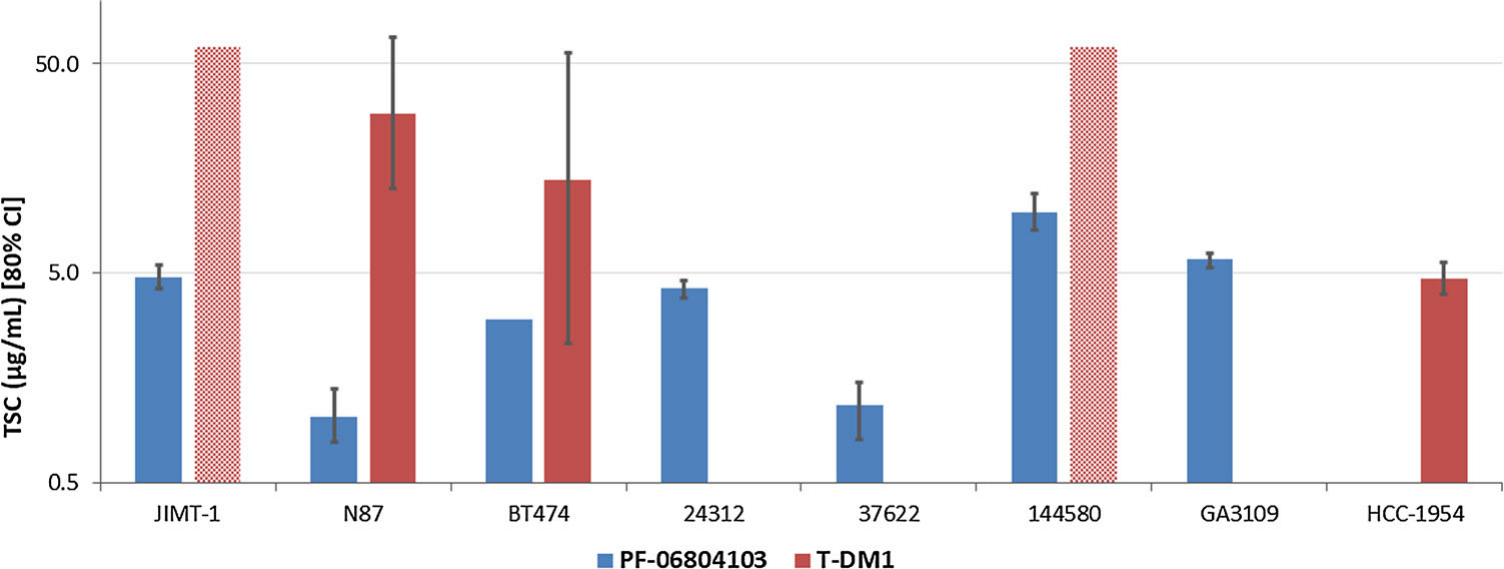
PF-06804103 and T-DM1 TSCs across mouse tumor xenograft models. T-DM1 was not responsive in JIMT-1 and 144,580 mouse tumor xenograft models (TSC values > 50 µg/mL). This is represented on the plot as hatched bars. The error bars represent 80% confidence intervals on TSC values

Minimal efficacious concentration (*Ceff*) in mouse xenograft models was defined as the concentration required for tumor stasis (TSC). PF-06804103 and T-DM1 TSC values with 80% confidence intervals across a range of CLX/PDX are shown in Fig. 2. Mean TSC of PF-06804103 was 4.3 µg/ml across 7 studies, with a range of 1.0–9.8 µg/mL. Mean TSC of T-DM1 was 15.8 µg/mL across 3 studies, with range of 4.7–29 µg/mL. JIMT-1 and 144580 mouse tumor models did not respond to T-DM1 and TSCs could not be determined in these models (> 50 µg/mL).

### Clinical PK modeling of T-DM1 using a TMDD model

**Fig. 3:**
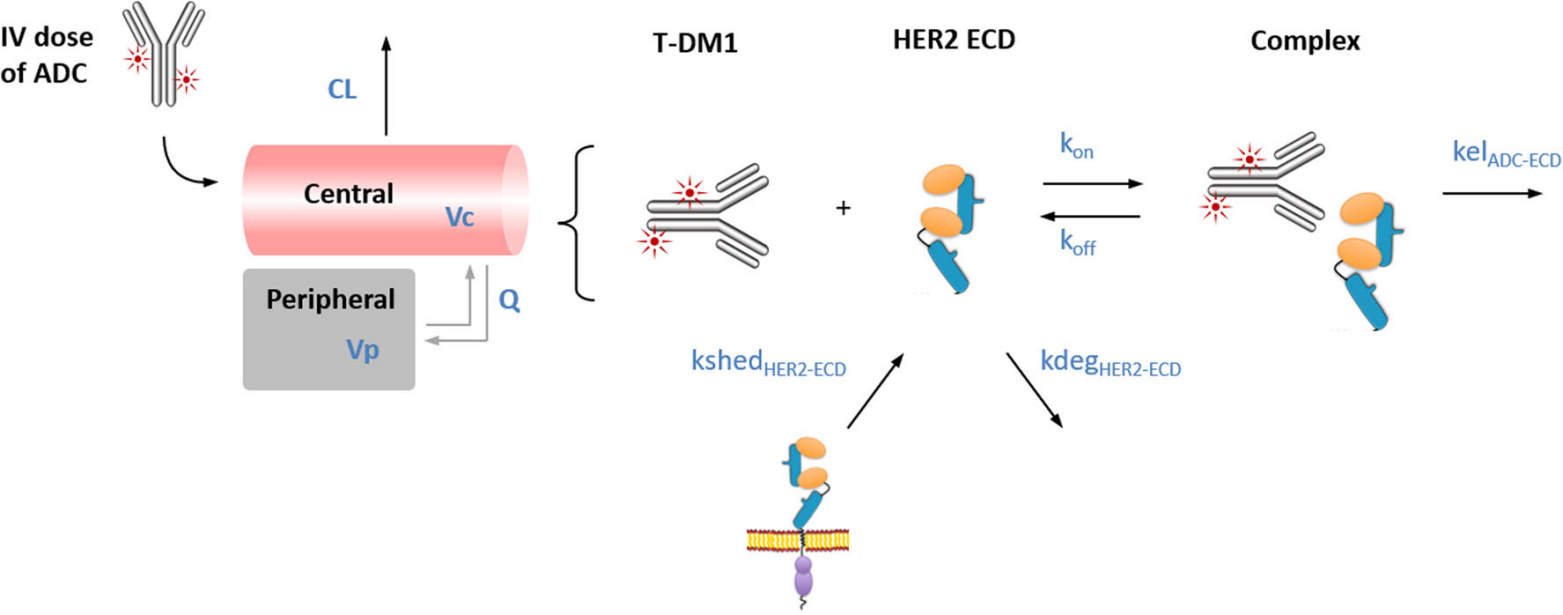
Target mediated drug disposition (TMDD) model used to describe clinical PK of T-DM1 and to predict clinical PK of PF-06804103. Please refer to Table 4 for description of the model parameters

**Fig. 4:**
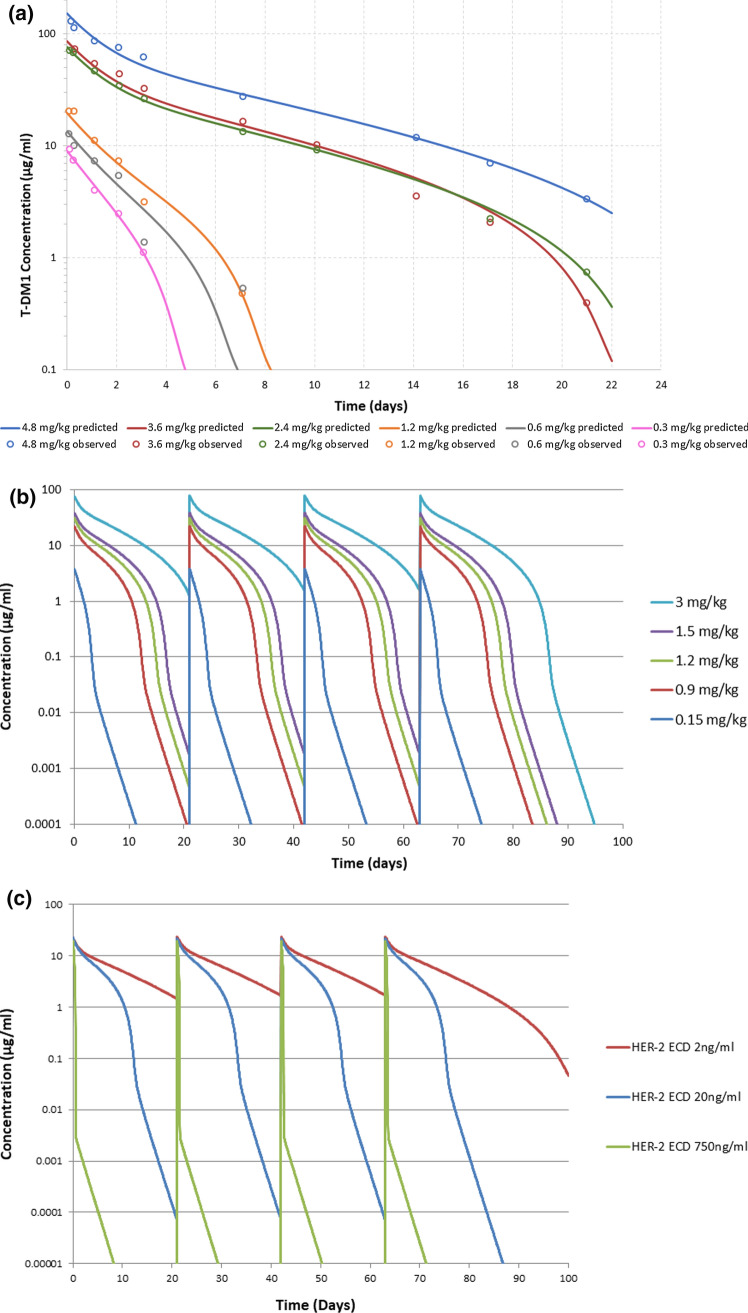
**a** TMDD model fit to T-DM1 Phase 1 clinical PK data (single dose administration) [24], **b** PK predictions for PF-06804103 using TMDD model (free drug concentrations) from 0.15- 3 mg/kg IV Q3W×4, **c** PK predictions for PF-06804103 following an IV dose of 1 mg/kg Q3W×4 to patients with low, medium and high HER2 ECD concentrations. These HER2 ECD concentrations are within the reported range for healthy females (low) and patients with advanced breast cancer (medium and high)

T-DM1 exhibits non-linear PK in the clinic, which is hypothesized to be due to binding to shed HER2 extracellular domain (ECD). A mechanistic TMDD model was developed to describe the clinical PK of T-DM1, which accounts for shedding of HER2 ECD into the serum, binding of T-DM1 to the ECD and subsequent clearance of the T-DM1-ECD complex (Fig. 3). The TMDD model parameters for T-DM1 are shown in Table 4 and the model fit to T-DM1 phase 1 clinical data [24] is shown in Fig. 4a.

**Table 4:** TMDD model parameters for T-DM1 in the clinic and predicted clinical PK of PF-06804103 following IV infusion of 1 h

Parameter (unit)	Description	T-DM1	Predicted PF-06804103
Vc (mL/kg)	Central compartment volume	37	38.1
CL (mL/day/kg)	Clearance	7.2	5.52
Vp (mL/kg)	Peripheral compartment volume	30	20.2
Q (mL/day/kg)	Inter-compartmental clearance	12	14.9
^a^K_D_ (nM)	HER2 binding affinity	0.1	
^*b*^*kshed *_*HER2*_*-*_*ECD*_ (nM day^−1^)	Rate constant for HER2 shedding	6.65	
*kdeg *_*HER2*_*-*_*ECD*_* (*day^−1^)	Rate constant for HER2 degradation	33.3	
*kel*_ADC-ECD complex_ (day^−1^)	Elimination rate constant of the HER2-ADC complex	32.6	
HER2 ECD (ng/mL)/(nM^c^)	Concentration of serum HER2 ECD	16–28/0.16–0.28	20/0.2

^a^K_D_ = *k*_*off*_/*k*_*on*_, ^b^*kshed *_*HER2*_*-*_*ECD*_ = *kdeg *_*HER2*_*-*_*ECD*_ x ECD (t = 0), ^c^Molecular weight of the HER2 ECD is 100 kDa

### Clinical PK projections for PF-06804103

The TMDD model developed for T-DM1 was applied to predict the human PK of PF-06804103. The 2- compartment linear IV PK parameters were scaled from cynomolgus monkey PK parameters (as described above, Table 1). The K_D_ was measured for PF-06804103, and all other parameters were estimated in the T-DM1 model. The predicted TMDD model IV PK parameters for PF-06804103 are shown in Table 4. The predicted PK profiles for PF-06804103 in the clinic following multiple dose administration of 0.15 mg/kg to 3 mg/kg IV Q3W × 4 are shown in Fig. 4b. Non-linear PK is predicted over this dose range with a predicted clearance of 33.6 mL/d/kg and elimination half-life of 1.0 day at the lowest simulated dose of 0.15 mg/kg, and a predicted clearance of 7.8 mL/d/kg with terminal half-life of 4.9 days at a dose of 3.0 mg/kg. These PK predictions are assuming a free drug assay. If a total assay is used (which measures free and bound drug) then the PK at each dose level would be as predicted for the high dose of 3.0 mg/kg.

Predicted PF-06804103 concentration versus time profiles following an IV dose of 1 mg/kg Q3W×4 in patients with low (2.0 ng/mL), medium (20 ng/mL) and high (750 ng/mL) serum HER2 ECD concentrations are shown in Fig. 4c. This figure indicates an inverse correlation between serum HER2 ECD concentration and PF-06804103 exposure. This relationship has also been observed for trastuzumab in clinical studies [6].

### Clinical PK/PD predictions for PF-06804103 and comparison with T-DM1

**Fig. 5:**
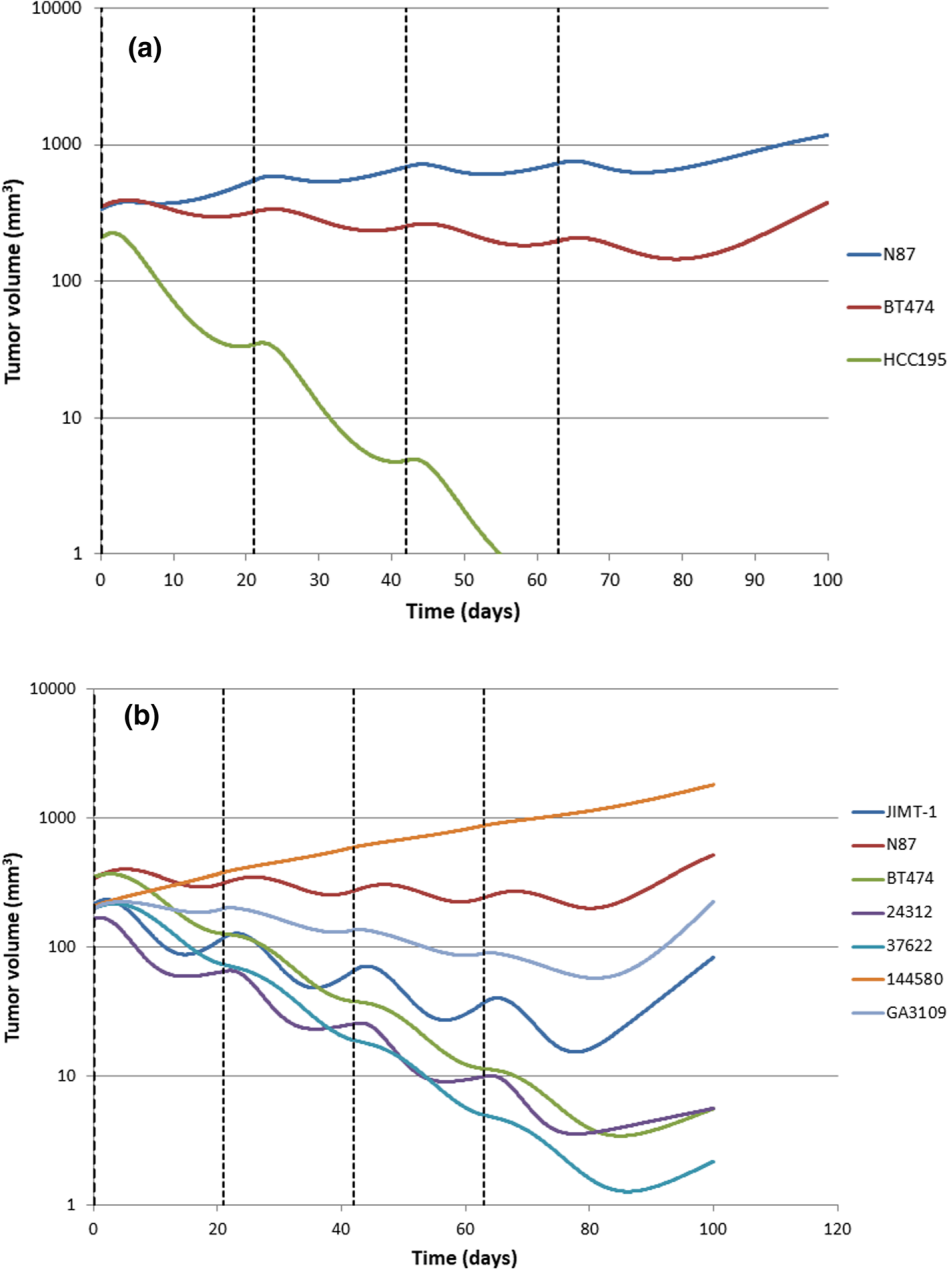
Translation of preclinical PK/PD model to the clinic for each tumor cell line model studied preclinically. Predicted efficacy of **a** T-DM1 following a 3.6 mg/kg Q3w dose and **b** PF-06804103 following a 1 mg/kg Q3w dose to cancer patients. The dashed vertical lines represent dosing times. The response in different cell lines is thought to be representative of response in individual patients

The clinical PK estimates from the TMDD model and the mouse PD model parameter estimates were integrated to simulate PF-06804103 and T-DM1 efficacy in the clinic. This approach assumes that ADC plasma concentrations are a good surrogate marker for the target site concentration that drives response and that mouse PD parameters translate directly to the clinic. Predicted efficacy of T-DM1 following 3.6 mg/kg Q3w×4 IV dose administrations and PF-06804103 following 1 mg/kg Q3w × 4 dose administrations are shown in Figs. 5a and b respectively. For T-DM1, N87 and BT474 models predict tumor stasis and HCC-195 predict tumor regression at 3.6 mg/kg Q3w in the clinic. For PF-06804103, 144580 predicts tumor re-growth, N87 predicts tumor stasis and JIMT-1, BT474, 24312, 37622 and GA-3109 all predict tumor regression at 1 mg/kg Q3w.

## Discussion

In this work we present the modeling and simulation strategy used to compare a new generation HER2 ADC (PF-06804103) with T-DM1, to ensure efficacy differentiation and as a rationale to pursue clinical development of PF-06804103. HER2 remains an exciting target to prosecute for oncology indications as it is clinically validated, with efficacy of HER2 targeted therapies established for breast and gastric patients that have HER2 amplification/ over-expression. In addition, recent data suggests that HER2 is over-expressed in a variety of other tumor types such as colon, bladder and biliary cancers, opening the door to new potential oncology indications for anti-HER2 therapies [28]. T-DM1 is a milestone drug which is standard of care second line treatment for patients with breast cancer and was the first ADC for the treatment of solid tumors. However, T-DM1 has limitations including moderate clinical activity (ORR 43.6% EMILIA and 31% in TH3RESA) [29] and Phase 3 failures (MARIANNE and GATSBY trials) [30, 31]. In addition, only high and homogeneously expressing HER2 tumors respond to T-DM1 [7]. The clinical activity of T-DM1 is also limited by intrinsic and acquired resistance. The mechanisms of resistance of T-DM1 are not completely understood, and the pharmacological complexity of this agent has confounded efforts to establish the clinically important mechanisms [15]. However, most evidence points to altered trafficking/ metabolism of T-DM1 and impaired lysine-MCC-DM1 mediated cytotoxicity as the predominant mechanisms of T-DM1 resistance in the clinic [15]. Loss of HER2 expression could also contribute to resistance, as has been proven for trastuzumab [32]. Also, evidence for mechanisms related to internalization, abnormal transit, lysosomal catabolism and drug efflux have been observed in non-patient derived experimental models [16, 33]. To help circumvent resistance, use of an alternative linker-payload in PF-06804103 would impede the T-DM1 resistance mechanisms that are specific to lysine-MCC-DM1 including impaired lysosomal degradation or enhanced efflux [16]. For all the reasons discussed above, novel differentiated HER2 therapies are required for the treatment of cancer.

### Modeling and simulation strategy

To quantitatively compare PF-06804103 and T-DM1 a translational PK/PD modeling and simulation strategy was implemented. This is a useful technique capable of integrating data generated from diverse test platforms in a mechanistic framework to describe exposure–response relationships [34]. The strategy described herein uses a mechanism-based tumor growth inhibition (TGI) model which integrates system parameters (tumor growth and initial tumor size) and drug effects (transduction rate, kill rate and potency). It is used to characterize TGI in mouse as a function of ADC concentration, making use of PK and PD data routinely generated for ADCs in the discovery phase. A population modeling approach was utilized to quantify variability in tumor cell growth across mouse tumor models. This is combined with a transduction model of tumor cell death driven by plasma ADC concentrations. The model can be translated to the clinic by incorporation of human PK and used to simulate dosing regimens required for tumor volume reduction in patients. It has been applied previously to study the clinical translation of T-DM1 and an anti-5T4 ADC (A1mcMMAF) [18]. This modeling approach differs from the larger quantitative systems pharmacology models that have been applied to ADCs to answer more complex mechanistic questions [35–37]. The level of model parsimony required depends upon the quantitative question asked [38]. In our case, the modeling question required comparison of 2 ADCs and a ‘fit-for-purpose’ modeling approach was applied, with the benefit that this could be easily re-applied to other ADCs or oncology drugs with a similar mechanism of action, such as mAbs or small molecule chemotherapeutics.

### Efficacy differentiation

To determine the preclinical efficacy of PF-06804103, studies were completed in a range of mouse tumor models, including models resistant to T-DM1. PDX and CLX were selected from different disease origins (breast, gastric and non-small cell lung cancer (NSCLC)). They also differed in HER2 expression levels. For example, N87 has 400,000 to 1 million HER2 receptors per cell, whereas JIMT-1 has 110,000 HER2 receptors per cell. To compare with T-DM1, some CLX/PDX were selected with susceptibility to both PF-06804103 and T-DM1. In addition, some ‘tougher’ models were selected such as JIMT-1, established from the pleural metastasis of a patient with breast carcinoma who had failed trastuzumab therapy, and PDX 144580, which was derived from a triple negative breast cancer patient.

TSC was used as a quantitative efficacy indicator to compare PF-06804103 and T-DM1 across models. It is defined as the concentration of the drug where the tumor is neither growing nor regressing and can be considered as the minimal concentration required for efficacy. TSC is a useful comparative metric as it combines information on the tumor growth pattern and the drug effect. TSC values for PF-06804103 were lower than for T-DM1 across the CLX/ PDX studied (Fig. 2). PF-06804103 was concluded to be more potent than T-DM1 across the mouse tumor cell lines studied and was efficacious in T-DM1 resistant models. Mechanistically, this makes sense as PF-06804103 has a cleavable linker which enables efficient intracellular release of membrane permeable payload and subsequent bystander killing. It is unknown whether the mechanisms of resistance to T-DM1 in animal models, that are overcome by PF-06804103, would directly translate to the clinical setting. However, alterations in lysine-mcc-DM1 mediated cytotoxicity appears to be a predominant mechanism of T-DM1 resistance in the clinic [15], which suggests that alternative therapies with different linker payloads may help overcome acquired T-DM1 resistance.

### Translation to human: PK

The first step in the clinical translation process was prediction of the clinical PK of PF-06804103. T-DM1 is known to exhibit non-linear PK in the clinic with increasing half-life and decreasing clearance values over the dose range studied in Phase 1 [24, 39]. For oncology drugs the size of the tumor is often not large enough to drive significant target mediated clearance. However, circulating soluble target can act as a sink for the drug and reduce the free levels of drug available to distribute to the tumor and bind to the target. The ECD of the HER2 receptor is shed from the cell surface and serum concentrations of HER2 have been shown to be higher in patients with metastatic breast cancer compared with healthy females [25]. Extremely high concentrations of HER2 ECD (approximately 1000 ng/mL) were observed in some patients with metastatic disease. For trastuzumab, high levels of serum HER2 ECD are associated with rapid CL and decreased benefit from trastuzumab therapy [6, 25, 40]. A TMDD model was developed for T-DM1 accounting for serum HER2 shedding, binding of T-DM1 to HER2 ECD and elimination of the T-DM1-HER2 ECD complex, in addition to the standard linear catabolic CL process (Fig. 3, Table 4). This model was shown to describe the non-linear CL observed for T-DM1 in Phase 1 studies (Fig. 4a, [24]). To test the model, it was used to predict PK of trastuzumab in a Phase 2 clinical study, where it was reported that a patient with high serum HER2 ECD exhibited vastly different PK to a patient with low serum HER2 ECD. Following IV administration of trastuzumab (250 mg loading dose, followed by 100 mg QW dosing), the patient with high HER2 ECD (> 700 ng/mL) showed rapid CL of trastuzumab resulting in steady state trastuzumab concentrations of approximately 4 µg/mL. The patient with low HER2 ECD (< 8.5 ng/mL) had steady state trastuzumab concentrations of approx. 70 µg/mL. The model was able to recapitulate the PK profiles with addition of only the reported HER2 ECD values and linear trastuzumab clearance (see Supplementary Fig. 2).

Since PF-06804103 is more potent than T-DM1 it may require lower doses for efficacy in the clinic. It was therefore considered important to predict the potential impact of non-linear clearance on the clinical PK of PF-06804103. The model developed for T-DM1 was applied to predict the PK of PF-06804103 in patients. The 2-compartment linear PK parameters were scaled from the cynomolgus monkey PK parameters for PF-06804103. The K_D_ for PF-06804103 binding to HER2 was included in the model. All other parameters, including shedding and degradation of the HER2-ECD and clearance of the PF-06804103-HER2 ECD complex were kept the same (Table 4). PF-06804103 is predicted to have similar PK to T-DM1.

### Translation to human: efficacy

Prior knowledge of the expected efficacy of an ADC in the clinic is desirable for optimal design of clinical trials and to ensure that an efficacious dose can be reached before the onset of dose limiting toxicities. In this analysis, preclinical PK/PD of PF-06804103 in mouse xenograft studies is translated to the clinic to compare predicted clinical efficacy with T-DM1. Prediction of clinical efficacy from mouse xenograft TGI is contentious and there is a long-held debate about their predictive capability [41–44]. Our thesis is that these studies contain rich information on the system and the effect of the drug. However, they are often not interpreted properly, and a systematic, rigorous quantitative method is required. To translate the preclinical PK/PD for PF-06804103 to human, the predicted human PK was incorporated, and it was assumed that mouse PD parameters translated directly to human. Since tumor doubling time is much slower in cancer patients (in the order of months) than in mouse experimental tumors (in the order of days), this represents a conservative approach and predictions that achieved stable disease (stasis) using mouse PK/PD parameters are assumed to be minimally efficacious in humans, achieving tumor regression. This method has been tested previously for T-DM1 and was shown to predict an efficacious dose of 2.4–4.8 mg/kg Q3W from modeling T-DM1 data from 3 mouse tumor models, which is consistent with the efficacious dose of 3.6 mg/kg Q3W [18]. An alternative approach would be to incorporate clinical tumor doubling times into the predicted clinical model, and this could be used for a more rigorous exploration of doses and regimens required for efficacy in specific patient populations. However, it is often difficult to obtain these rates, due to absence of placebo data. As such, a fit-for purpose approach was taken which is useful to compare between PF-06804103 and T-DM1, and has been shown to successfully predict clinical efficacious dose of T-DM1 [18].

Translation of PF-06804103 to the clinic predicts efficacy at lower doses than T-DM1 (Fig. 5a and 5b). These figures illustrate the benefit of studying several mouse tumor models to characterize efficacy and translate to the clinic. Depending on their individual characteristics and susceptibilities, different CLX and PDX tumor models predict a range of effects from complete response to tumor regrowth. An alternative approach to determining efficacy in mouse models was reported for PF-06804103 and T-DM1 [8]. They evaluated in vivo efficacy in a panel of HER2 + gastric and NSCLC PDX and completed a waterfall analysis, using RECIST criteria to define overall response rate (ORR). The NSCLC PDX were designated HER2^1+^ to HER2^2+^ and the gastric PDX were designated HER2^1+^ to HER2^3+^ by immunohistochemistry. In the panel of gastric cancer PDX models, PF-06804103 and T-DM1 had an ORR of 3/3 (100%) and 0/3 (0%), respectively. In the panel of NSCLC cancer PDX models, PF-06804103 and T-DM1 had an ORR of 8/9 (89%) and 1/10 (10%), respectively.

In conclusion, modeling and simulation strategies were used to demonstrate that a new generation HER2 ADC (PF-06804103) is a potentially exciting new therapy which differentiates from T-DM1 in its preclinical efficacy profile. PF-06804103 had a lower Ceff (TSC) in mouse models using CLX/PDX with both high and low HER2 expression and was efficacious in T-DM1 resistant models. Clinical PK of PF-06804103 is predicted to be similar to T-DM1 and non-linear across doses. The mouse PK/PD models were translated to the clinic and predicted superior efficacy compared to T-DM1. As a result, PF-06804103 is projected to provide benefit in HER2+ indications in the clinic.

## Electronic supplementary material

Below is the link to the electronic supplementary material.

Supplementary file1 (DOCX 396 kb)
