# Supplementary material for: Use of translational modeling and simulation for quantitative comparison of PF-06804103, a new generation HER2 ADC, with Trastuzumab-DM1
Source: J Pharmacokinet Pharmacodyn. 2020 Jul 24;47(5):513–26. doi: 10.1007/s10928-020-09702-3 (PMC7520420; doi:10.1007/s10928-020-09702-3)

**Supplementary Materials**

**Supplementary Fig. 1** Goodness of fit plots for PF-06804103 PK/PD modeling in 3 CLX and 4 PDX xenograft mouse models

Plots shown include: (i) visual predictive checks of tumor volume (mm3) data and model prediction versus time at each dose level. The magenta band represents the 95% prediction distribution and (ii) observations versus model predictions using the population and individual parameters compared to line of unity.

| JIMT-1:   | JIMT-1:   |
| --- | --- |
| N87:   | N87:   |
| BT474:   | BT474:   |
| 24312:  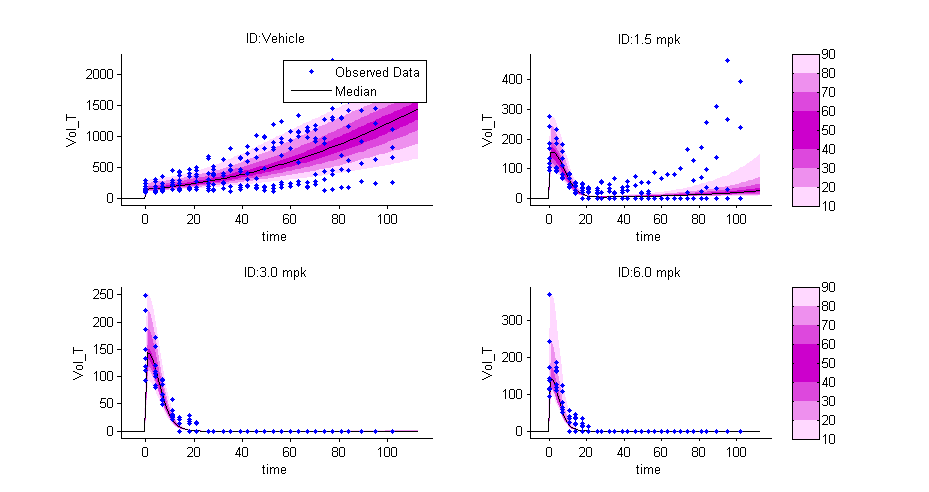 | 24312:  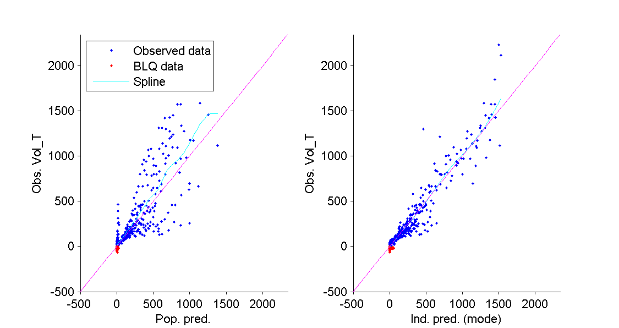 |
| 37622:  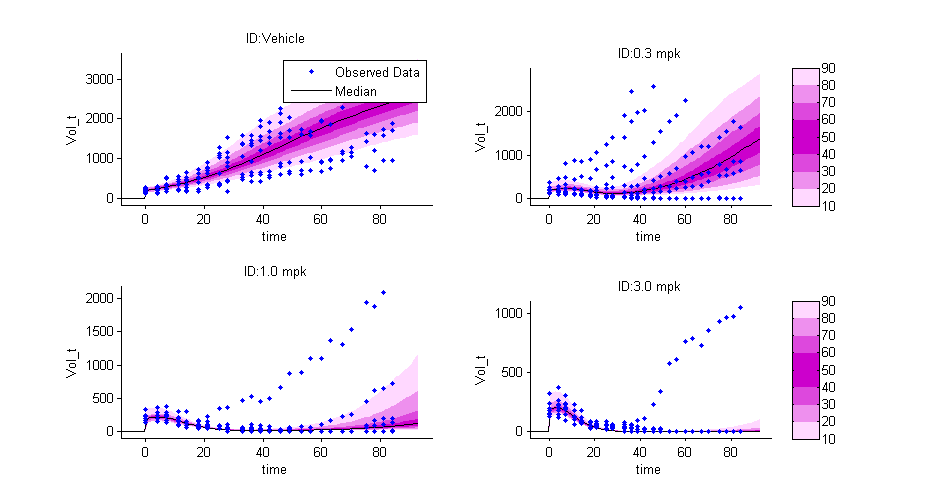 | 37622:  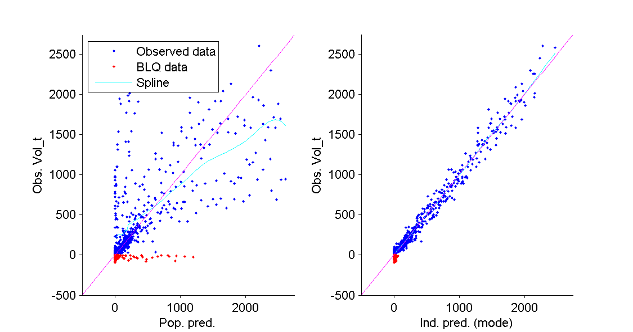 |
| 144580:  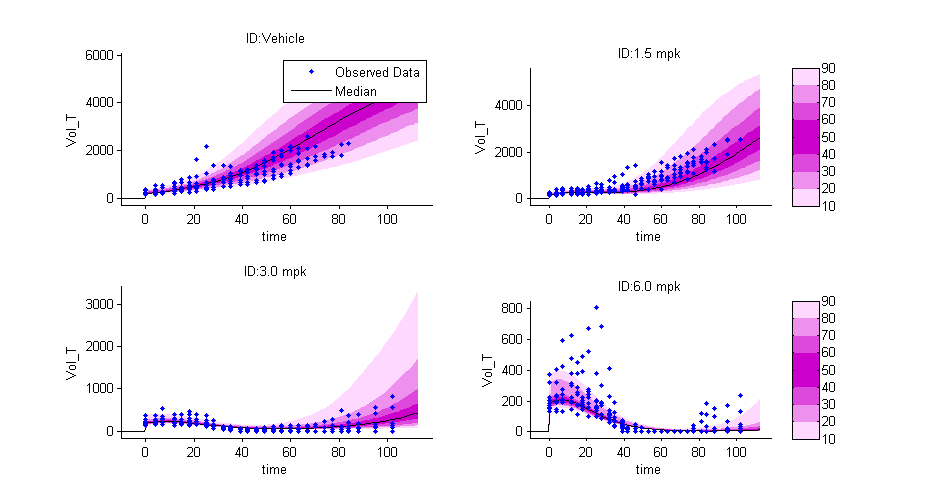 | 144580:  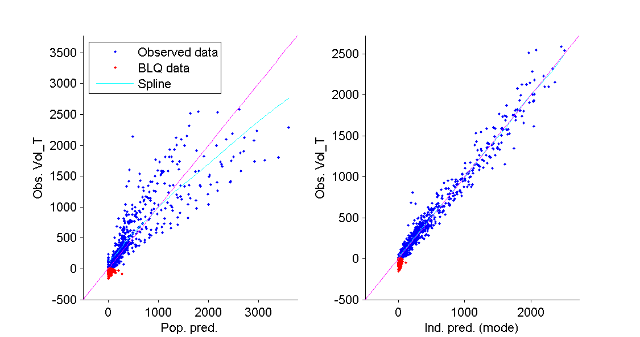 |
| GA3109:  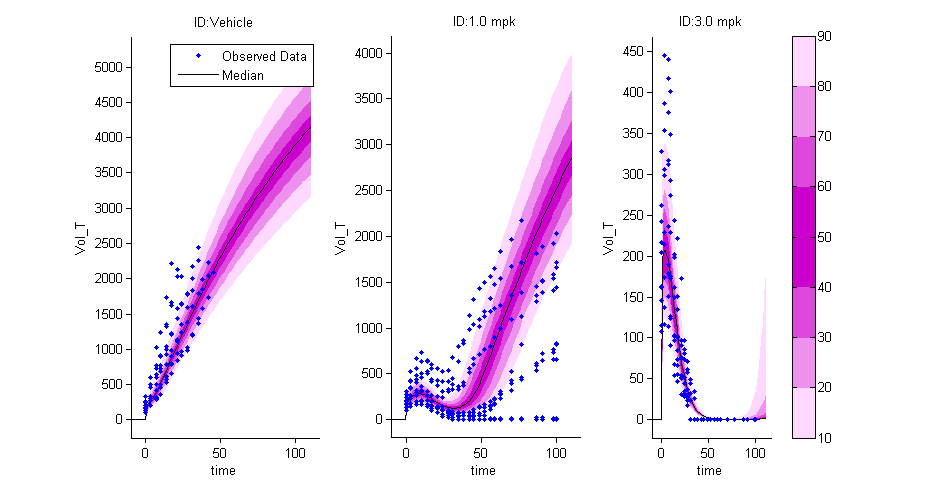 | GA3109:  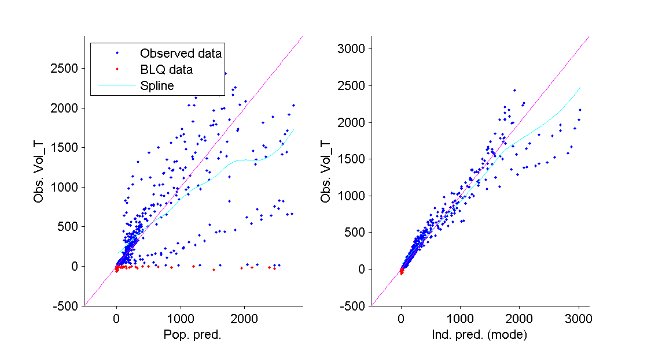 |

**Supplementary Fig. 2** Validation of TMDD model by application to predict PK from a Phase II study [6] where discrepant PK was observed following trastuzumab administration to a patient with **(a)** low HER2 ECD (<8.5 ng/mL) and a patient with **(b)** high HER2 ECD concentration (>750 ng/ml).

**Trastuzumab dose was 250mg IV loading dose, 100mg IV thereafter.** Symbols represent data digitized from [6]. Lines represent TMDD model predictions. A CL value of 0.23 mL/h/kg was used for trastuzumab. In (a) HER-2 ECD concentration was set to 2 ng/mL and in (b) HER2 ECD concentration was set to 750 ng/ml. All other parameters were kept the same.

**(a)**


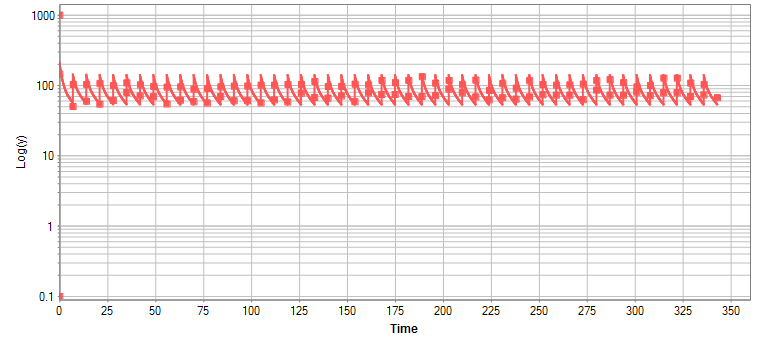


**(b)**


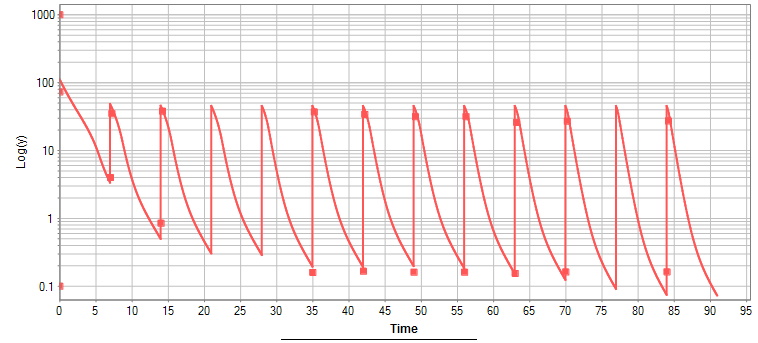

Supplement: Supplementary file 1 — Supplementary file1 (DOCX 396 kb) [file 10928_2020_9702_MOESM1_ESM.docx]
